# Bio-Inspired PHM Model for Diagnostics of Faults in Power Transformers Using Dissolved Gas-in-Oil Data

**DOI:** 10.3390/s19040845

**Published:** 2019-02-18

**Authors:** Huanyu Dong, Xiaohui Yang, Anyi Li, Zihao Xie, Yuanlong Zuo

**Affiliations:** 1 College of Information Engineering and college of Qianhu, Nanchang University, Nanchang 330031, China; 6002115114@email.ncu.edu.cn (H.D.); 6101116067@email.ncu.edu.cn (A.L.); 2 College of Information Engineering, Nanchang University, Nanchang 330031, China; 6101116073@email.ncu.edu.cn (Z.X.); 416114317077@email.ncu.edu.cn (Y.Z.)

**Keywords:** power transformer PHM, bat algorithm, BP neural network, fault diagnosis

## Abstract

Prognostics and Health Management (PHM) is an emerging technique which can improve the availability and efficiency of equipment. A series of related optimization of the PHM system has been achieved due to the growing need for lowering the cost of maintenance. The PHM system highly relies on data collected from its components. Based on the theory of machine learning, this paper proposes a bio-inspired PHM model based on a dissolved gas-in-oil dataset (DGA) to diagnose faults of transformes in power grids. Specifically, this model applies Bat algorithm (BA), a metaheuristic population-based algorithm, to optimize the structure of the Back-propagation neural network (BPNN). Furthermore, this paper proposes a modified Bat algorithm (MBA); here the chaos strategy is utilized to improve the random initialization process of BA in order to avoid falling into local optima. To prove that the proposed PHM model has better fault diagnostic performance than others, fitness and mean squared error (MSE) of Bat-BPNN are set as reference amounts to compare with other power grid PHM approaches including BPNN, Particle swarm optimization (PSO)-BPNN, as well as Genetic algorithm (GA)-BPNN. The experimental results show that the BA-BPNN model has increased the fault diagnosis accuracy from 77.14% to 97.14%, which is higher than other power transformer PHM models.

## 1. Introduction

Prognostics and Health Management (PHM) is an emerging technology related to system life-cycle support that enables conspicuously lower expenses on maintenance, usage, and support, and enhances the reliability and availability of the system. It has attracted a great deal of attention in recent years [1,2,3]. It focuses on evaluating the health of equipments according to the related information from abnormal sensing, diagnosis, prognosis, and health management [4]. Several works have attempted to predict the Remaining Useful Life (RUL) of equipment in the system and to prevent ultimate catastrophic failure [5].

Regarded as a complex system with numerous equipment, the power grid has become more intelligent with the continuous promotion of technologies [6]. In general, a power system is composed of many electric transmission and distribution plants the failure of which may lead to the failure of the system, so that fault identification of components such as a power transformer which is a critical component, constitutes an important stake.

A power transformer, with the task of electric transmission and distribution, is a significant equipment within a smart grid [7]. Therefore, prognostics and health management of its potential faults play an important role in ensuring the safe operation of the power system. The faults of a power transformer can be divided into interior and exterior fault modules [8]. In an oil-immersed transformer, any harmful operating conditions to the power transformer may lead to decomposition of insulating materials and release of gaseous decomposition products dissolved in oil [9]. Correspondingly, Dissolved Gas-in-oil Analysis (DGA) as well as its improved approaches including the Rogers Ratios, and IEC Ratio are widely used as an efficient method to analyze the characteristic gases in an oil-immersed transformer and its fault [10,11]. However, traditional DGA methods have their limitations that cannot produce a reasonable fault result by the simple combination of a few ratio coding [12].

To tackle the restrictions mentioned above, artificial intelligence (AI) techniques based on machine-learning theory have been applied to build PHM models including artificial neural network (ANN) [13] and support vector machine (SVM) [14]. Back-propagation neural network (BPNN) is an optimized algorithm of ANN, namely, the error in BPNN can be thrown back after the feed-forward process [15]. BPNN obeys the gradient descent rule, which highly depends on initial weight and threshold. If the parameter selection is improper, it may cause a slow convergence speed and easily relapse into local extremes. A newly metaheuristic method, bat algorithm (BA), was proposed by Yang in 2010 [16]; it is adopted to optimize the initial value of BPNN to obtain a better performance. Recent research has demonstrated that BA outperforms other swarm intelligence optimization methods like Particle swarm optimization (PSO) and Genetic Algorithm (GA) [17]. Therefore, a PHM model based on the theory of machine learning that is combined with BA and BPNN is proposed in this paper. Then, it is employed in a power transformer to test its reliability.

This paper is recommended by the SDPC 2018 committee; concerning the limitation in the previous paper, several modifications have been made in this paper including updating the rule of population initialization and the visualization of some results in the classification process from real oil-immersed power transformer data collected by sensors in a power grid. The major contributions are listed as follows: (1) To validate the BA for the power transformer fault diagnosis problem and utilitie the MSE function to test it. (2) In order to avoid falling into local optima, this paper is based on the chaos strategy proposes modified BA (MBA), which introduces the sinusoidal map instead of the random initialization to modify the population of the standard BA. (3) The evaluation of the proposed machine learning-based PHM model is based on real operational data collected from an oil-immersed transformer in a power grid. The corresponding result proves the better performance of the proposed model for transformer fault diagnosis compared to other traditional PHM methods.

This paper is organized as follows: Section 2 describes the basic methodologies of modified BA and BPNN and modifies the standard BA. The proposed MBA-BPNN PHM model is introduced in Section 3, and the structure flow of the proposed PHM model is drawn in this part. Section 4 employs MBA-BPNN to power transformer fault diagnosis, then tests the model with a DGA dataset from a real oil-immersed power transformer to demonstrate the effectiveness. In addition, Section 4 also compares a series of newly intelligent PHM models to prove that MBA-BPNN has better performance in reaching target values. Section 5 provides several remarks and future modifications in the proposed model.

## 2. Description of Basic Methods

### 2.1. Modified Bat Algorithm

#### 2.1.1. Standard Bat Algorithm

Bat algorithm is a novel swarm intelligence optimization algorithm, which was proposed by Yang [18]. It is based on the advanced property of bat species; after emitting ultrasonic pulses into their surroundings, they make use of echolocation to search for prey and to sense distance. The sound pulse varies with distance. In particular, the whole process can be formulated as a multi-objective optimization method. Initially, BA is made up of a great deal of candidate solutions (swam of bats) which fly at a certain velocity. Meanwhile, the individual in each has its own location and is able to adjust the velocity and its location according to the optimal solution [19]. For simplicity, the echolocation characteristics of the artificial bats are represented as follows [18]:

- All the bats use echolocation property to sense the distance and are able to distinguish the difference between prey and surroundings.
- Based on the fixed frequency *f* that fulfills the condition $f_{min}$ ≤ *f* ≤ $f_{max}$, different loudness $A_{0}$ and various wavelength, bats fly randomly with velocity $v_{i}$ at position $x_{i}$ correspondingly. They are able to adjust the wavelength (or frequency) of the pulses and the pulse emission rate rϵ[0,1] depending on proximity.
- The loudness $A_{0}$ ranging from a small predefined value $A_{min}$ to $A_{0}$.

According to the assumptions, the whole process consists of global and local search phases. During the global search phase, the movement at iteration *t* of each individual bat *i*th is updated as follows:

(1) $$x_{i}^{t}=x_{i}^{t-1}+v_{i}^{t}$$

(2) $$v_{i}^{t}=v_{i}^{t-1}+\left(x_{i}^{t}-x_{*}\right)f_{i}$$

(3) $$f_{i}=f_{min}+\left(f_{max}-f_{min}\right)\beta$$

Here, $x_{*}$ is the best solution at present, and the random variable βϵ[0,1], which represents the uniform distribution. There are some similarities in the update process of the velocities and positions of bats between PSO and BA [20].

Thereafter in the local search phase, a new solution $x_{i}^{t}$ for bat *i* is generated according to the random walk procedure:

(4) $$x_{i}^{t}=x_{*}+\varepsilon A^{t}$$

where $x_{*}$ is a solution selected among the current optimal solutions, ε is a random number between [−1,1]. $A^{t}=\langle A_{i}^{t}\rangle$ is the current mean loudness of all the bats.

Furthermore, the loudness gradually decreases and the pulse emission rate increases as bats get closer to their prey. With the iterations proceeding, the loudness and the pulse rate are updated by:

(5) $$A_{i}^{t}=\alpha A_{i}^{t-1}$$

(6) $$r_{i}^{t}=r_{i}^{0}\left(1-exp\left(-\gamma t\right)\right)$$

Here α is a constant ranging from 0 to 1, another constant γ is larger than 0. As the iteration number tends to infinity,

$$A_{i}^{t}\to 0,r_{i}^{t}\to r_{i}^{0}$$

#### 2.1.2. Modification of Initial Population

According to the description of original BA, the random initialization of bats leads to the lack of population versatility, which can easily cause local optima [21]. Thus, in order to improve the ability and to overcome the problem, a modification of the initial population inspired by the chaos strategy [22] is proposed in [23]. The dynamic characteristics of the chaos strategy allow the algorithm-generated solution to be fully diversified, potentially reaching each mode in a multi-objective environment [23], which is the main reason to apply the chaotic initialization of the bat population.

In the modification of the initial population of standard BA, the random initialization of the bat population is replaced by chaotic maps. Compared with other chaotic maps [24], The Sinusoidal map has the best performance, which can be described specifically as follows:

(7) $$x_{i+1}=\alpha x_{i}^{2}sin\left(\pi x_{i}\right)$$

All the *N* bats fly in *d*-dimensional space, they are randomly distributed in a vector $z_{i}$, and its iteration obeys Equation (8). Correspondingly, the location *x* of the bat *i* can be calculated by:

(8) $$x_{i}=\frac{x_{maxd}-x_{mind}}{2}z_{i}+\frac{x_{maxd}+x_{mind}}{2}\left(i=1,2,\cdots ,N\right)$$

The pseudo-code of modified BA is shown as Algorithm 1. 

**Algorithm 1:** Pseudo-code of the modified BA.

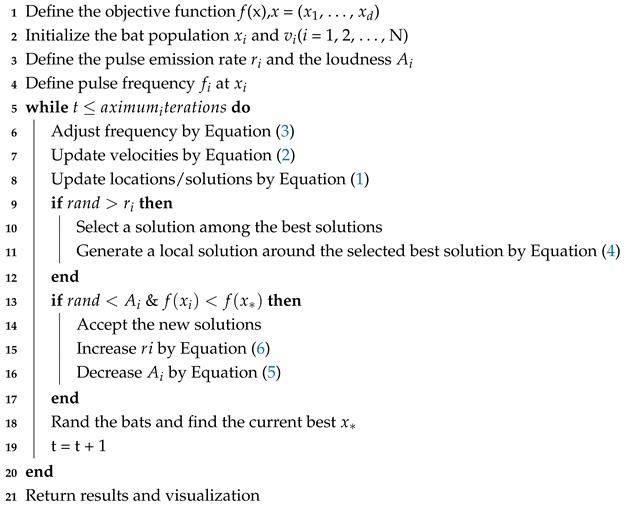



### 2.2. Back-Propagation Neural Network

**Figure 1:**
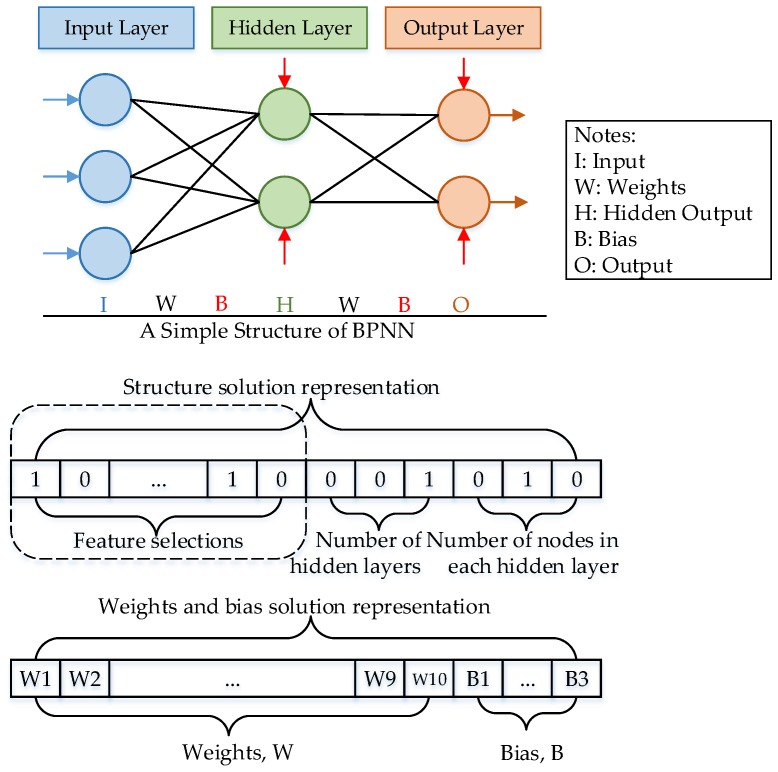
Back-Propagation neural network model structure.

Back-propagation Neural Network (BPNN) is an erroneous reverse transmission algorithm [25] with the property of nonlinear mapping between any input and output as well as self-learning, which makes it extensively used for the diagnosis of transformer faults [26]. It is a local search algorithm based on the input-hidden-output layer structure to speed up the process of gaining a weight matrix and threshold. The classification and forecasting process consists of feed-forward and back-propagation. The Back-Propagation neural network model structure can be seen in Figure 1. The whole iteration process is expressed as follows:

(9) P(k+1)=P(k)−ηΔf(P(k))

#### 2.2.1. Feed-Forward

After recording the input value vector *x*, the activation $a^{l}$ in the input layer *l* can be computed in a simple and compact vectorized form:

$$a^{l}=f\left(\omega^{l}a^{l-1}+b^{l}\right)\left(l=2,3,\cdots ,L\right)$$

here, $\omega^{l}$ and $b^{l}$ are the weight and the bias between the ${\left(l-1\right)}^{th}$ and the ${\left(l\right)}^{th}$ layer.

To set the corresponding activation, this paper uses the Sigmoid function as the transfer function, which has good performance in solving the relationship between nonlinear input and output [27]:

(10) $$\sigma \left(x\right)=\frac{1}{1+e^{-x}}$$

The quadratic error criterion function of sample *n* is *C*:

(11) $$C=\frac{1}{2n}\sum_{x}{∥y\left(x\right)-a^{L}\left(x\right)∥}^{2}$$

#### 2.2.2. Back-Propagation

While reaching at the layer *L*,the output error $\delta^{L}$ can be calculated by

(12) $$\delta^{L}={▽}_{a}C⊙\sigma^{{}^{\prime }}\left(z^{L}\right)$$

${▽}_{a}C$ contains the rate of *C* changing. ⊙denotes the entry-wise product of two vectors. Subsequently, the error in the next layer is

(13) $$\delta^{l}=\left({\left(\omega^{l+1}\right)}^{T}\right)\delta^{l+1})⊙\sigma^{{}^{\prime }}\left(z^{l}\right)$$

where ${\left(\omega^{l+1}\right)}^{T}$ is the transpose for the ${\left(l+1\right)}^{th}$ layer. $⊙\sigma^{{}^{\prime }}\left(z^{l}\right)$ is Hadamard product within interval [0,1].

The back-propagation network obeys the steepest descent algorithm to adjust the weights and the thresholds, and the threshold can be calculated as follows:

(14) $$\frac{\partial C}{\partial b_{j}^{l}}=\delta_{j}^{l}$$

Any weight in the network is:

(15) $$\frac{\partial C}{\partial \omega_{jk}^{l}}=a_{k}^{l-1}\delta_{j}^{l}$$

The error is obtained through the activation function in layer *l* by combining above equations; the problem of slow convergence rate of BPNN is solved correspondingly [28].

## 3. The Proposed MBA-BPNN PHM Model

According to the description of conventional BPNN, the initial value is of great significance to the descent method. Once the selection of the related value is highly biased, BPNN falls into the local minimum. BA has strong global search and fast convergence speed capabilities, which is adopted to optimize the initial value of BPNN. In this section, this paper combines two sorts of bio-inspired algorithms, modified BA with BPNN, to complement each other. The modified BA is utilized to optimize the structure of the BPNN, the matrix weight of which is initialized firstly with the bat population. Following that, the weight and threshold are passed to BPNN to start the training state; correspondingly, the best solution among the results of the neural network is selected out. Here, a random walk of BPNN is imbedded to search new solutions and each new acceptable solution should be updated by the best solution if it is optimal. This process is accomplished if the last iteration is reached or if MSE (Mean Square Error) is achieved. Thus, the optimal values of the weight matrix are found. The combination of modified BA and BPNN can increase the learning ability and make up for the disadvantage of slow convergence speed [29].

This paper applies MSE between the factual results $Y_{i}$ and expected output $O_{i}$ to evaluate the performance of MBA-BPNN, which is calculated by:

(16) $$f=\frac{1}{n}\sum_{i=1}^{n}{∥Y_{i}-O_{i}∥}^{2}$$

where *n* is the number of training sample.

**Figure 2:**
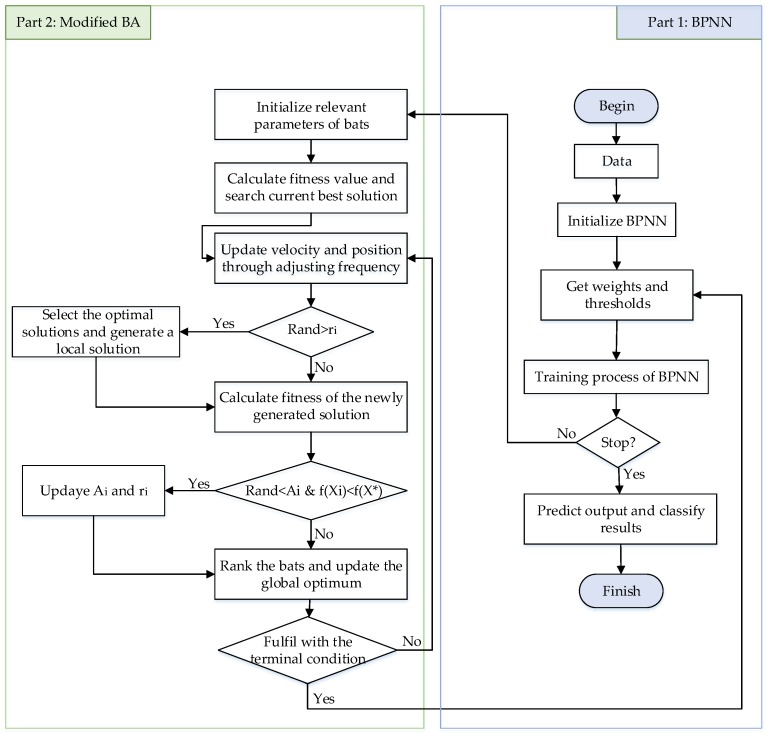
Flowchart of MBA-BPNN model.

The flowchart of proposed to improve BA-BPNN is drawn in Figure 2 and the main steps are discussed below:

Step 1: Randomly load the attribute data and obey the characteristics mentioned in Section 2.2.

Step 2: Initialize the related parameters of the BPNN including the node number of input, hidden and output layers.

Step 3: Initialize the bat population characteristics, which are positions $x_{i}$, velocity $v_{i}$, loudness $A_{i}$, pulse emission rate $r_{i}$ as well as frequencies $f_{i}$.

Step 4: Set the current optimal solution $x^{*}$.

Step 5: Get the global new solution for each individual bat of the population by BPNN.

Step 6: Compare whether the new solution is better than the current solution. The new solution is accepted if the fitness is better; then, update the location of a bat *i* and loop to Step 9; if not, return to Step 7.

Step 7: Calculate the new solution according to the equations and generate a random number *rand*, if rand>r; generate a local solution around the best solution.

Step 8: If $rand<A\&\&f\left(x_{i}\right)<f\left(x^{*}\right)$, accept the new solution, update the loudness $A_{i}$ and pulse emission rate $r_{i}$.

Step 9: Rank the bats and update the global optimal solutions.

Step 10: Determine whether the last iteration is reached or MSE is achieved, then, end the process; if not, return to Step 5.

## 4. Validation of the Proposed PHM Model for Power Transformer Fault Diagnosis

In this section, the proposed PHM model is employed in a power transformer, specifically, it focuses on the relationship between the typical gas-in-oil-immersed transformer and actual fault type.

**Figure 3:**
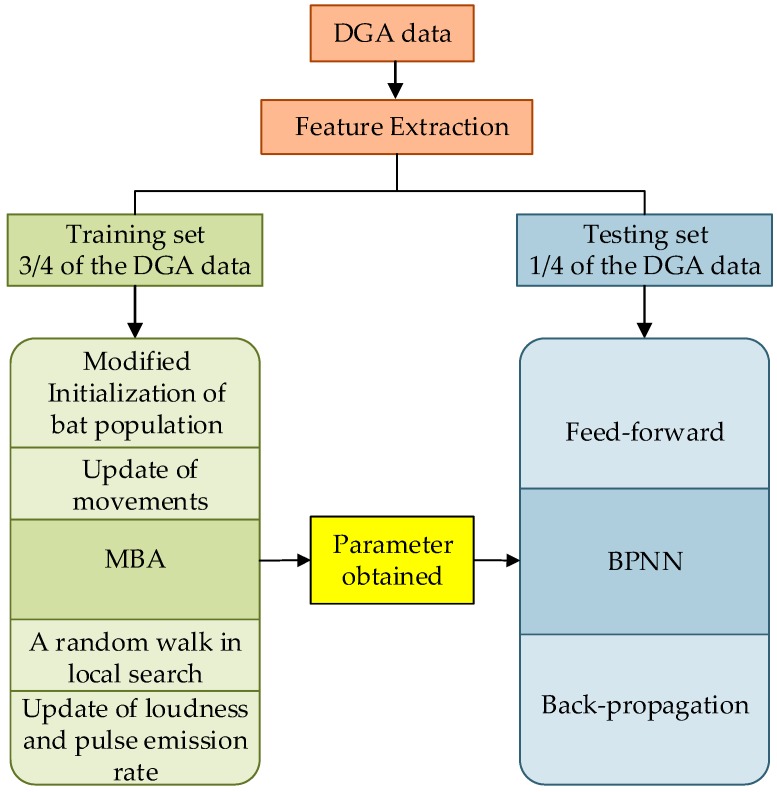
MBA-BPNN PHM model for power transformer fault diagnosis.

Each set of characteristics gas is mapped to a sort of fault type, which is called classification process. Following that a new set is produced by the power transformer, it can be predicted the related fault type based on the MBA-BPNN model as shown in Figure 3. Those two parts of the process achieve the goal of PHM that the prognostic and health management of equipment. The whole process of fault diagnosis is simplified as four parts including collection and preprocessing of data, segmentation of dataset, training process of BPNN, as well as comparison between test set output and training set output, which is shown as follows.

### 4.1. The Initialization of BPNN

In this paper, 109 sets of DGA data are collected from power transformer in State Grid of Jiangxi Province in China and used for the validation of the proposed MBA-BPNN 163 model for power transformer fault diagnosis. Those power transformers operate in 220 kVA of voltage and 50 Hz of power frequency. The temperature is 25 ${}^{\circ }$C and the humidity is 50%. Each data group consists of the characteristics gases ($H_{2}$, $CH_{4}$, $C_{2}H_{6}$, $C_{2}H_{4}$, $C_{2}H_{2}$) and its actual fault type. The normalization process samples are 165 to [−1,1] which are used as the input dataset.

According to *IEC60599*, the fault type is classified into five sorts including local discharge, middle-low temperature overheat, high temperature overheat, low-energy discharge and high-energy discharge. In addition, humidification fault is a complex type, which is challenging to differ based on the analysis result [30]. It should be decided comprehensively combined with exterior check and other experimental result. Thus, power transformer faults in this paper discussed are middle-low temperature overheat (LT), high temperature overheat (HT), low-energy discharge (LD), high-energy discharge (HD), and partial discharge (PD), as the output, the corresponding code is shown in Table 1.

Depending on the input and output set, the structure of BPNN is set as 3-30-5, namely, the input layer has three nodes, the hidden-layer has 25 nodes, the output layer has five nodes. As the description of BPNN in Section 2, the weights of BPNN are 3×30+30×5=240 and the threshold are 30+5=35. So, the total number of parameters optimized by MBA is 275.

**Figure 4:**
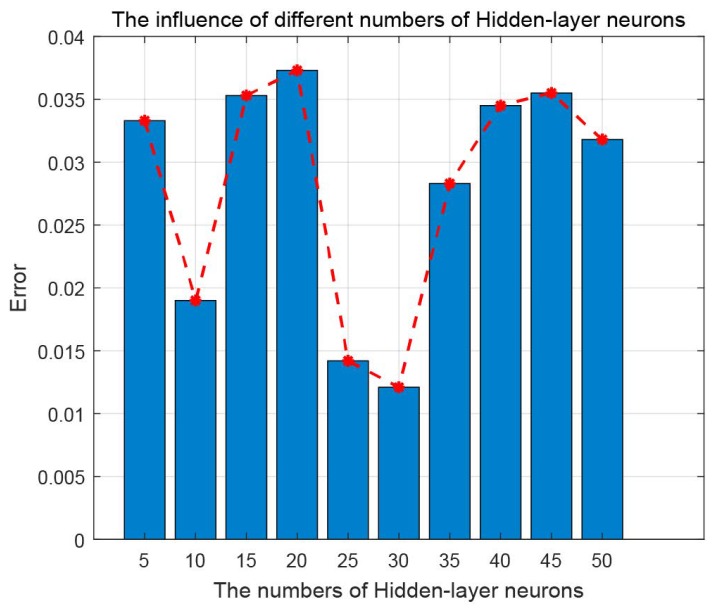
The error of MBA-BPNN PHM model as the number of Hidden-layer neurons increasing.

The validation of the proposed MBA-BPNN model is shown in Figure 4. This paper sets different number of hidden-layer neurons ranging from 5 to 50. Ceteris paribus, the error varies along with the numbers of hidden-layer neurons. When the hidden-layer neuron is 30, the error reaches the smallest 0.013.

### 4.2. The Training Process of BPNN

The training part is a process in which weight and threshold, the key parameters of the structure of BPNN, are adjusted continuously in order to decrease the error as much as possible. The training target of the proposed model is 0.01, the learning rate is set at 0.2, the iteration number is 1000. During the training process, the weight can be corrected in order to get the BP neural network error smaller.

In BA, the bat population size is set to 20 and the number of generations is 50. The initial loudness is set to 0.5, the initial pulse emission rate set at 0.5.

### 4.3. Classification and Prediction of Bat-BPNN PHM Model

In the experiments, 3/4 of the DGA data (79 objects) is chosen as the training set, and 1/4 of the DGA data (30 objects) is used as the testing set. The experiment is carried out in the environment of Matlab and related results are shown in Table 2. The other three PHM methods, traditional BPNN, GA-BPNN and PSO-BPNN, based on the same DGA data are compared in order to prove that the proposed method is valid. It is apparently to see MBA-BPNN outperform the traditional BPNN (77.14%), GA-BPNN (88.57%), PSO-BPNN (91.43%) with higher diagnosis accuracy 95.22%. Moreover, BA-BPNN is simpler than those optimized methods with less memory usage.

**Figure 5:**
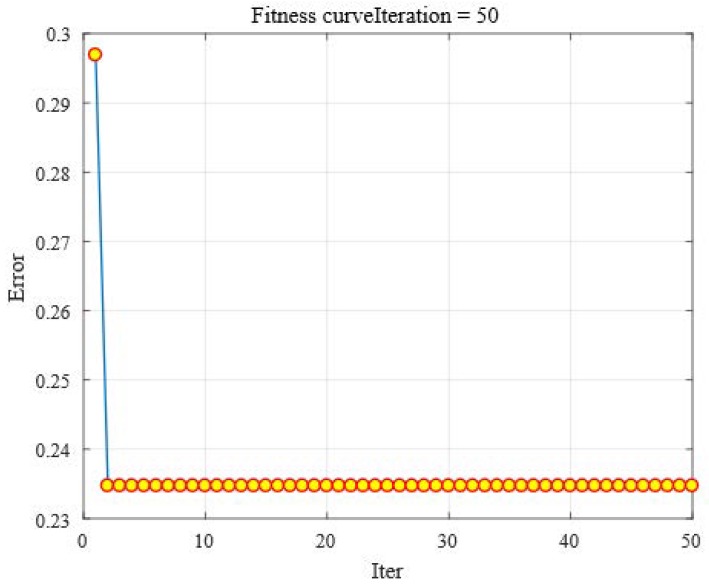
The fitness curve of MBA-BPNN PHM model.

To analyze the bat behavior of MBA-BPNN, a fitness function is employed to calculate the error in this paper; the curve of fitness improves (in Figure 5) as the number of iterations increases. That fitness value drops rapidly from 0.297 to 0.235 within two generations, which shows high efficiency of global and local optimization of the MBA-BPNN PHM model, but following that the MBA-BPNN falls into a local extremum point, the proposed PHM model continues to search for the optimal solutions, while the fitness is always staying at 0.235. Furthermore, MBA can minimized the training error in a lower number of set iterations, and succeed to optimize the performance of BPNN in power transformer fault diagnosis.

In terms of MSE that represents the performance of prediction, MBA-BPNN has outstanding performance compared to other PHM models, as is shown in Table 3. The proposed PHM model has both the smallest MSE on training and test samples compared with traditional BPNN, GA-BPNN as well as PSO-BPNN, which approaches the expected value better.

**Figure 6:**
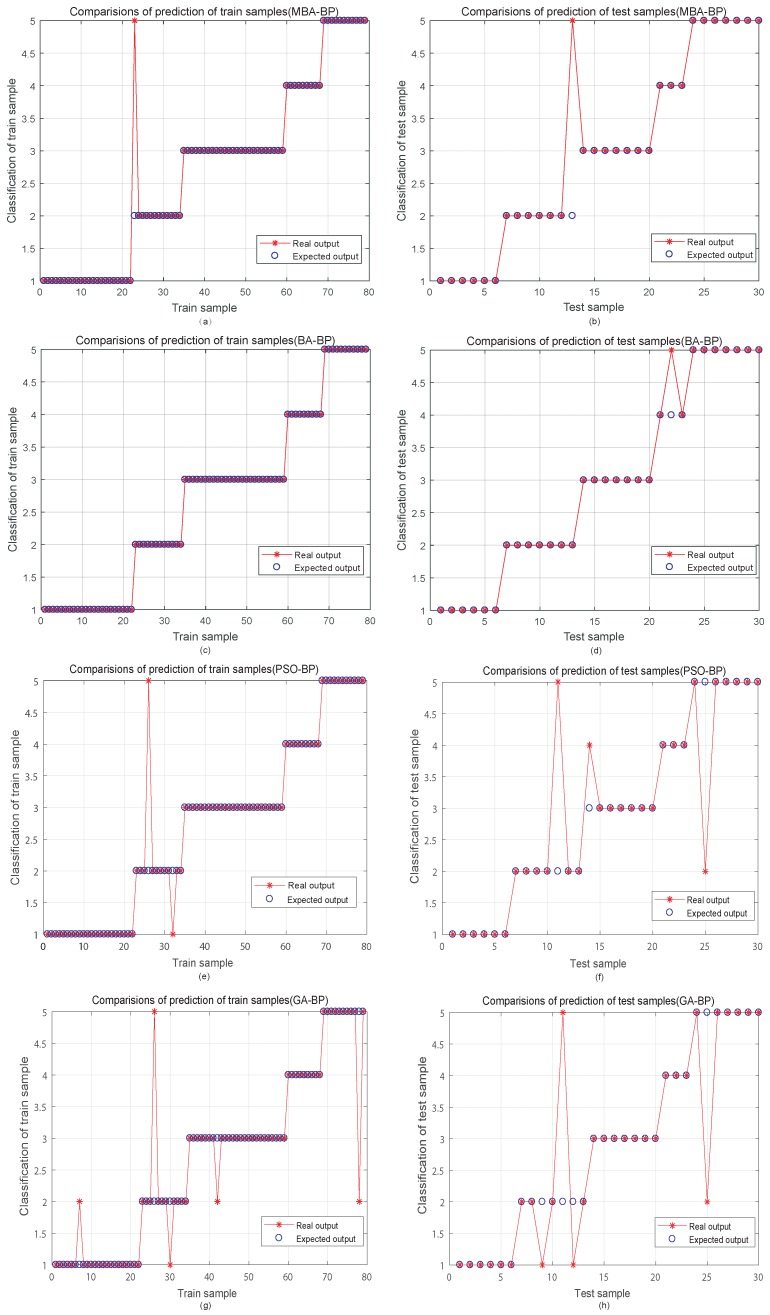
Classification results of several PHM models. (**a**,**c**,**e**,**g**) are the classification results of train sample for different methods, respectively. (**b**,**d**,**f**,**h**) represent the classification results of test sample for different methods, respectively.

Figure 6 shows the ability of prediction among a few PHM models, the more points overlapping indicates a better prediction effect. Compared with other optimized models, BA outperforms other bio-inspired algorithms in predicting the output. It improves the ability of the trained model to predict that the output is closer to the actual value after acquiring the DGA data. Based on the same data sample, MBA-BPNN can easily avoid the problem of over-fitting. Therefore, the MBA-BPNN PHM model is an efficient tool for power transformer fault diagnosis.

## 5. Conclusions

Prognostic and health management of oil-immersed transformers are vital to ensure the stable reliability. A series of relevant diagnostic models including BPNN based on the relationship between characteristic gas and typical fault type has been established in recent years, but the accuracy of classification and prediction cannot meet actual needs.

As a sort of data-driven PHM model, BPNN highly depends on its parameters of structure (weight and threshold), which are set by experience in the initialization. This paper employs a bio-inspired BA to BPNN, which can make them complement each other. Here, BA is excellent in a global search to find the best parameters of BPNN; then, BPNN utilizes the data collected by sensors to predict the next stage of the power transformer. While standard BA is easy to fall into local optima, a chaos strategy is applied to make BA fully adopt the dynamic environment and get diverse solutions. Results in Section 4 demonstrate that the accuracy for fault diagnosis has been increased. Compared with other PHM models, MBA-BPNN has properties of getting closer to the target value and fast speed of convergence.

However, humidification fault cannot be identified by the proposed PHM model due to the similarity between both. Future modifications to the MBA-BPNN PHM model are to tune the other parameters mentioned in Section 2.1 based on the theory of machine-learning. In particular, the updating rule of location should be adjusted by a fixed vector, which can avoid the numeral operation executing in just one physical dimension. In addition, the procession rule of data collected by the sensors can be replaced by an IEC-Three Ratio, which can be coded in another form.

In conclusion, the MBA-BPNN model is not only adopted for power transformer fault diagnosis, but is also suitable for other classification and prediction problems in prognostic and health management with better performance and high accuracy.

## Figures and Tables

**Table 1:** Output code of various fault types.

No.	Fault Type	Fault Type
1	HT	00001
2	LT	00010
3	HD	00100
4	LD	01000
5	PD	10000

**Table 2:** Fault Diagnosis Accuracy of MBA-BPNN and other PHM models [31].

Fault Type	MBA-BPNN	BA-BPNN	PSO-BPNN	GA-BPNN	BPNN
1	100%	100%	100%	100%	100%
2	85.71%	100%	85.71%	57.14%	100%
3	100%	100%	85.71%	100%	85.71%
4	100%	66.67%	100%	100%	100%
5	100%	100%	85.71%	85.71%	0%
Average accuracy	97.14%	93.33%	91.43%	88.57%	77.14%

**Table 3:** Comparison of MSE of MBA-BPNN and other PHM models.

Fault Type	MBA-BPNN	BA-BPNN	BPNN	GA-BPNN	PSO-BPNN
MSE_Train Sample	0.0054	0.0092	0.0330	0.0196	0.0124
MSE_Test Sample	0.0190	0.0287	0.1571	0.0378	0.0484
